# Real‐World Outcomes of Baricitinib and Ritlecitinib in Refractory Alopecia Areata: Response Predictors and Relapse After Discontinuation or Dose Reduction

**DOI:** 10.1111/1346-8138.70241

**Published:** 2026-03-31

**Authors:** Rina Hayashi, Saori Takamura, Tomoo Fukuda

**Affiliations:** ^1^ Department of Dermatology Saitama Medical Center, Saitama Medical University Saitama Japan

**Keywords:** alopecia areata, baricitinib, ClinRO, JAK inhibitor, ritlecitinib, SALT score

## Abstract

Baricitinib and ritlecitinib are newly approved systemic Janus kinase (JAK) inhibitors for severe, refractory alopecia areata (AA). Although clinical trials have demonstrated their efficacy, real‐world evidence remains limited, particularly regarding predictors of response and outcomes following treatment interruption or dose tapering. To evaluate the real‐world effectiveness and safety of baricitinib and ritlecitinib in adults with refractory AA, and to identify factors associated with treatment response and relapse. We retrospectively reviewed 65 adults with refractory AA treated at a tertiary center (baricitinib 4 mg/day, *n* = 50; ritlecitinib 50 mg/day, *n* = 15). Scalp regrowth was evaluated using the Severity of Alopecia Tool (SALT) up to Week 52, with response defined as SALT ≤ 20 (≥ 80% scalp coverage). Predictors of Week 36 response were assessed in the baricitinib cohort using multivariable logistic regression. Relapse outcomes after treatment discontinuation or dose reduction were documented with extended follow‐up. Adverse events (AEs) were recorded. At Week 36, 42% of patients receiving baricitinib achieved SALT ≤ 20, comparable to the ritlecitinib group (41.7%). In the baricitinib cohort, disease duration < 2 years and baseline eyebrow loss (ClinRO score 3) independently predicted Week 36 response (*p* < 0.05). All four responders who discontinued baricitinib relapsed (mean 14.7 weeks). Among 13 patients who reduced the dose to 2 mg/day, relapse occurred in 6/12 evaluable cases (mean 24.2 weeks). Most AEs were mild, and no serious events occurred. Baricitinib demonstrated meaningful real‐world efficacy, particularly in patients with shorter disease duration and baseline eyebrow involvement. Relapse was common after treatment cessation or dose tapering, supporting the need for close monitoring for approximately 3 months after discontinuation and at least 6 months following dose reduction. These findings may inform patient selection and long‐term treatment planning, and highlight the importance of further prospective studies to optimize JAK inhibitor management in AA.

## 1 Introduction

Alopecia areata (AA) is a chronic, immune‐mediated hair loss disorder characterized by sudden, nonscarring patchy hair loss, with a lifetime prevalence of roughly 2% globally. In Japan, recent survey‐based estimates place the prevalence of AA between ~1.5% and 2.2%, and notably, over one‐third of diagnosed individuals are not receiving any treatment, underscoring a significant unmet need [1]. Beyond its dermatologic manifestations, severe AA imposes a profound psychosocial burden. A large population‐based study in the U.K. reported that patients with AA have significantly higher rates of depression and anxiety than controls (adjusted hazard ratios ~1.3–1.4), along with increased work disability (56% greater likelihood of work absence and 82% higher odds of unemployment) [2]. These observations highlight the importance of improving treatment outcomes to alleviate both clinical and psychological burdens.

Conventional therapies for AA–including corticosteroids (topical, intralesional, or systemic), contact immunotherapy, and broad immunosuppressants–often result in inconsistent or temporary improvement, particularly in extensive or chronic cases. Until recently, reliable systemic treatment options were lacking, and moderate‐to‐severe AA was often refractory to therapy [3]. The therapeutic landscape has changed with the development of Janus kinase (JAK) inhibitors, which target key immune pathways implicated in AA pathogenesis. Cytokines such as interferon‐γ and interleukin‐15 drive cytotoxic T‐cell–mediated follicular attack through the JAK–STAT signaling cascade [4]. A landmark preclinical and early clinical study demonstrated that pharmacologic blockade of this pathway could reverse AA, first in a murine model and subsequently in humans [5]. These discoveries led to clinical trials of JAK inhibitors, ultimately resulting in approved therapies. Baricitinib, a JAK1/2 inhibitor, became the first oral systemic agent approved for severe AA, followed by ritlecitinib, a selective JAK3/TEC‐family inhibitor, which has shown efficacy in phase III trials and is now approved in several countries for adolescents and adults with severe AA [6, 7].

Pivotal trials have demonstrated that JAK inhibitors can induce substantial hair regrowth in a proportion of patients with severe AA. In two randomized phase III trials (BRAVE‐AA1 and BRAVE‐AA2), baricitinib 4 mg once daily achieved the Severity of Alopecia Tool (SALT) ≤ 20 response (≥ 80% scalp coverage) by Week 36 in ~35%–40% of participants–significantly outperforming placebo [8]. Similarly, in a phase IIb/III trial, 23%–25% of Asian patients receiving ritlecitinib 50 mg daily achieved ≥ 80% scalp coverage by Week 24, compared with ~2% in the placebo group [9]. Despite these advances, real‐world evidence remains limited. Clinical trial populations may differ from routine practice due to strict inclusion criteria, and several clinically relevant questions remain unanswered–for example, how these agents perform in long‐standing or refractory disease, what factors predict treatment response, and what outcomes can be expected after treatment discontinuation or dose tapering.

To address these gaps, we conducted a real‐world observational study of adults with refractory AA treated with baricitinib or ritlecitinib in routine clinical practice. Our objectives were to evaluate drug effectiveness and safety at 36 weeks, identify clinical predictors of response, and characterize relapse outcomes following treatment discontinuation or dose reduction after regrowth. Through this work, we aim to inform optimal patient selection, maintenance strategies, and treatment sequencing for JAK inhibitors in severe AA–particularly within the Japanese clinical context, where real‐world evidence remains scarce.

## 2 Subjects and Methods

### 2.1 Patients and Study Design

This retrospective observational study included adult patients with refractory AA who received baricitinib or ritlecitinib at the Department of Dermatology, Saitama Medical University General Medical Center between 2022 and 2025. “Refractory” AA was defined as extensive and/or relapsing disease characterized by high SALT scores or chronically recurrent alopecia in which prior conventional therapies had failed to induce adequate improvement. Patients aged ≥ 15 years were eligible for inclusion, consistent with the minimum approved age for baricitinib use in Japan.

Among the 65 enrolled patients, 50 were treated with baricitinib and 15 with ritlecitinib. In the ritlecitinib group, 60% had previously received baricitinib and were transitioned due to insufficient clinical response. Baricitinib was initiated at 4 mg once daily and could be tapered to 2 mg/day at the discretion of the treating dermatologist in responders, while ritlecitinib was administered at the approved dose of 50 mg/day. Patients were followed at regular intervals to document scalp regrowth, eyebrow/eyelash changes, and adverse events as part of routine clinical practice.

This study was conducted in accordance with the Declaration of Helsinki and was approved by the institutional review board of Saitama Medical University (Approval No. 2025087). Because of the retrospective nature of the study using anonymized clinical data, informed consent was obtained via an opt‐out process.

### 2.2 Outcome Measures

The primary efficacy outcome was scalp hair regrowth at Week 36. Disease severity was evaluated using the SALT score, where SALT 100 indicates complete scalp hair loss and SALT 0 indicates full coverage. Treatment response was defined as achieving SALT ≤ 20 (≥ 80% scalp regrowth) at Week 36. Secondary outcomes included SALT score changes at Weeks 24 and 52, eyebrow/eyelash regrowth, and durability of treatment response.

Eyebrow and eyelash involvement was assessed using a clinician‐reported outcome (ClinRO) scale ranging from 0 to 3 (0 = no loss, 1 = mild loss, 2 = moderate loss, 3 = complete loss). Safety was evaluated by recording all adverse events documented in medical records during treatment, and their frequency and severity were summarized for each treatment group.

### 2.3 Data Collection and Analysis

Clinical and demographic variables collected included age, sex, disease duration, previous treatments, baseline SALT score, baseline eyebrow/eyelash ClinRO scores, treatment regimen (dose and duration), SALT trajectory over time, achievement and timing of treatment response, and post‐response management (continuation, tapering, or discontinuation). For patients who discontinued or reduced dosage, the time to relapse was recorded. All adverse events were reviewed and categorized.

To identify predictors of treatment response, multivariate logistic regression was performed in the baricitinib cohort with achievement of SALT ≤ 20 by Week 36 as the dependent variable. Independent variables included age, sex, AA duration, previous therapies, baseline SALT score, and eyebrow/eyelash ClinRO scores. Correlations between changes in SALT scores and eyebrow/eyelash ClinRO scores were assessed using Spearman's rank correlation coefficient, given the ordinal nature of the ClinRO scale and nonparametric distribution of the data. Statistical analyses were performed using JMP version 18 (SAS Institute, Cary, NC, USA), and *p* < 0.05 was considered statistically significant. In addition to the primary endpoint (SALT ≤ 20 at Week 36), relative improvement endpoints were evaluated. SALT50 and SALT75 were defined as ≥ 50% and ≥ 75% relative improvement from baseline SALT score at Week 36, respectively, calculated as (Baseline SALT–Week 36 SALT) / Baseline SALT ≥ 0.50 or ≥ 0.75. Multivariable logistic regression analyses using the same covariates as the primary model were performed to explore predictors of SALT50 and SALT75 achievement.

## 3 Results

### 3.1 Patient Characteristics at Baseline

A total of 65 patients with refractory AA were included in the analysis, comprising 50 treated with baricitinib and 15 with ritlecitinib. Baseline demographic and clinical characteristics are summarized in Table 1.

**TABLE 1:** Baseline demographic and clinical characteristics of patients with refractory alopecia areata treated with baricitinib or ritlecitinib.

Characteristic	Baricitinib (*N* = 50)	Ritlecitinib (*N* = 15)
Age, years	46.0 (36.8–53.5)	53.0 (39.0–64.0)
Sex (female/male)	56.0%/44.0%	66.7%/33.3%
Duration from initial AA onset to treatment initiation, years	6.0 (0.08–66.0)	9.0 (1.0–56.0)
Duration from most recent AA episode to treatment initiation, years	3.0 (0.08–24.0)	8.0 (0.5–56.0)
Duration from initial AA onset to treatment initiation < 2 years	32.0% (*n* = 16)	20.0% (*n* = 3)
Duration from most recent AA episode to treatment initiation < 2 years	54.0% (*n* = 27)	33.3% (*n* = 5)
Baseline SALT score	80 (50–100)	60 (50–100)
Baseline SALT score (SALT 95–100/≤ 94)	34.0% (17)/66.0% (33)	26.7% (4)/73.3% (11)
**Alopecia phenotype**		
Alopecia totalis	62.0%	60.0%
Alopecia universalis	38.0%	40.0%
Baseline ClinRO (Eyebrow)score (0/1/2/3)	36.0%/16.0%/16.0%/32.0%	46.7%/13.3%/33.3%/6.7%
Baseline ClinRO (Eyelash)score (0/1/2/3)	40.0%/12.0%/22.0%/26.0%	46.7%/6.7%/33.3%/13.3%
Comorbid atopic dermatitis (presence/absence)	44.0%/56.0%	40.0%/60.0%
History of Intravenous Corticosteroid Pulse Therapy (presence/absence)	26.0%/74.0%	13.3%/86.7%
Non‐responders to Baricitinib	N/A	60.0%

*Note:* This table summarizes baseline demographic features, disease chronicity, alopecia severity, eyebrow and eyelash hair involvement, and prior systemic treatment exposure in patients receiving baricitinib or ritlecitinib. Variables include duration from initial disease onset and from the most recent episode to treatment initiation, Severity of Alopecia Tool (SALT) score, alopecia phenotype, Clinician‐Reported Outcome (ClinRO) scores for eyebrows and eyelashes, prior JAK inhibitor exposure, and comorbid atopic dermatitis. The structured format enables direct cross‐group comparison of initial disease burden and phenotypes, providing essential context for interpretation of subsequent therapeutic outcomes. Data are presented as median (interquartile range [IQR]) or percentage, unless otherwise indicated.

Abbreviations: AA, alopecia areata; ClinRO, clinician‐reported outcome; SALT, severity of alopecia tool.

### 3.2 Disease Duration and Chronicity

The duration from initial AA onset to treatment initiation tended to be longer in the ritlecitinib cohort. The median time from initial onset was 6.0 years in the baricitinib group and 9.0 years in the ritlecitinib group. Similarly, the median duration of the most recent AA episode was 3.0 years and 8.0 years, respectively.

When early disease was defined as < 2 years in duration, 32.0% (16/50) of baricitinib‐treated and 20.0% (3/15) of ritlecitinib‐treated patients met this threshold based on initial onset. For the most recent episode, early disease (< 2 years) was observed in 54.0% (27/50) and 33.3% (5/15) of patients, respectively, indicating comparable recent disease activity between both cohorts.

### 3.3 Severity of Alopecia

Marked baseline severity was observed in both groups. SALT ≥ 80 was documented in 54.0% of the baricitinib cohort and 46.7% of the ritlecitinib cohort. Complete eyebrow loss (ClinRO score 3) was more common in the baricitinib group (30.0%) compared with the ritlecitinib group (6.7%), while complete eyelash loss occurred in 24.0% and 13.3%, respectively. SALT ≥ 95 was documented in 34.0% (17/50) of the baricitinib cohort and 26.7% (4/15) of the ritlecitinib cohort.

### 3.4 Prior Treatments

In the ritlecitinib cohort, 60% had previously received baricitinib and were switched due to inadequate response. Other previous therapies included topical/intralesional corticosteroids, systemic immunosuppressants, or other oral JAK inhibitors, consistent with the definition of refractory AA.

### 3.5 Baricitinib Efficacy of Hair Regrowth

**FIGURE 1:**
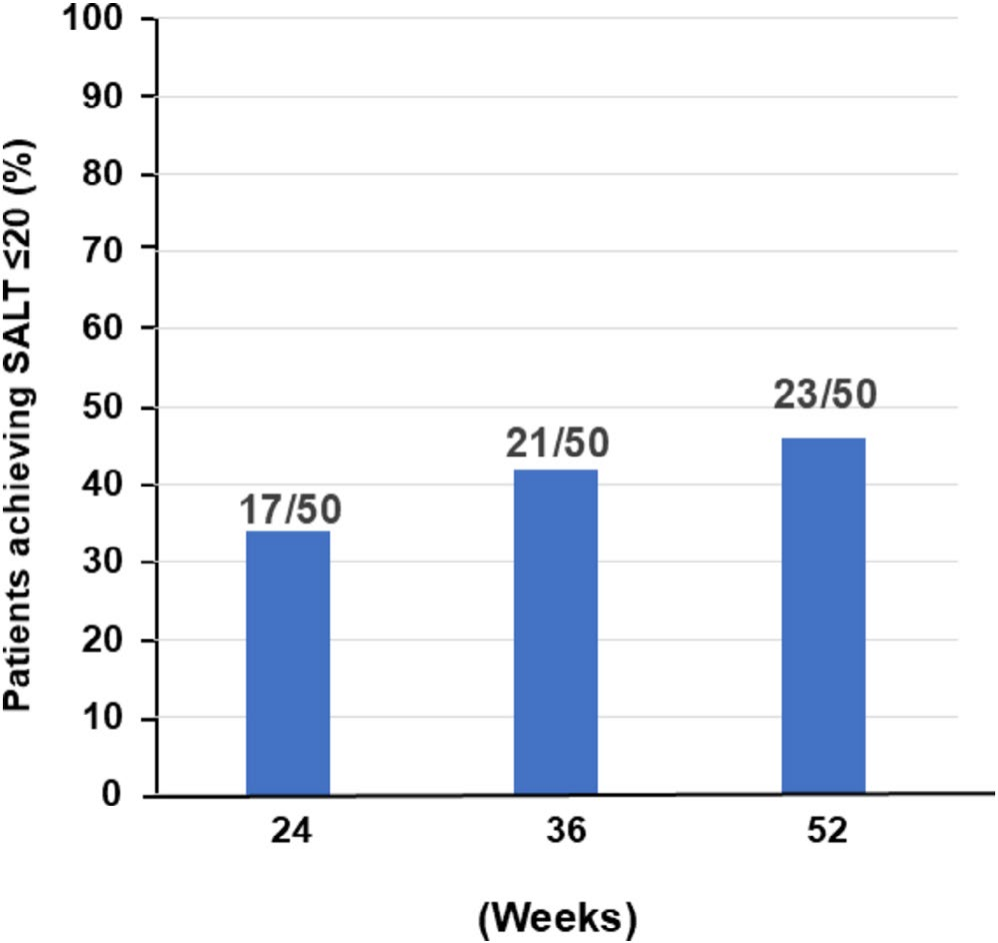
Time‐course of scalp hair regrowth during baricitinib therapy. This figure illustrates the proportion of patients with severe alopecia areata who achieved clinically meaningful scalp hair regrowth during baricitinib treatment. Treatment response was defined as attaining a Severity of Alopecia Tool (SALT) score ≤ 20, corresponding to ≥ 80% scalp coverage. Response rates improved progressively over time, with 34.0% at Week 24, 42.0% at Week 36, and 46.0% at Week 52. These findings indicate gradual and sustained regrowth under continuous therapy in real‐world clinical practice, highlighting the value of ongoing treatment to capture both early and late responders.

We assessed SALT ≤ 20 response over time in each group. In the baricitinib group, the SALT ≤ 20 response rate was 34.0% (17/50 patients) at Week 24, increasing to 42.0% (21/50) by Week 36. By Week 52, the response rate further rose slightly to 46.0% (23/50). These results indicate that around one‐third of patients had at least 80% hair regrowth by 24 weeks of baricitinib treatment, and this proportion increased to nearly half by 1 year (Figure 1).

### 3.6 Predictors of Treatment Response

We analyzed factors associated with a favorable response to baricitinib at Week 36 (Table 2). Based on established evidence from phase III clinical trials of baricitinib, achievement of a SALT score ≤ 20 at Week 36 was selected as the primary outcome measure. In the multivariate analysis of the baricitinib‐treated cohort, two independent predictors of achieving a treatment response by Week 36 were identified (all *p* < 0.05): a shorter duration from the initial onset of alopecia areata (< 2 years) and complete eyebrow loss at baseline (ClinRO eyebrow score of 3) (Table 2). These findings indicate that patients with more recent disease onset and those presenting with complete eyebrow involvement were more likely to achieve substantial hair regrowth with baricitinib therapy. In contrast, female sex, a baseline SALT score < 95, complete eyelash loss at baseline (ClinRO eyelash score of 3), and a history of intravenous corticosteroid pulse therapy were not significantly associated with treatment response. Additional analyses using relative improvement thresholds demonstrated consistent findings. Baseline SALT score **<** 95 was significantly associated with achievement of SALT50 at Week 36 and showed a similar directional association with SALT75. Baseline complete eyebrow loss also demonstrated consistent trends across these relative improvement endpoints. Although the number of patients achieving higher improvement thresholds limited statistical power in multivariable models, no additional independent predictors emerged beyond those identified for the primary SALT ≤ 20 outcome. Detailed results are presented in Tables S1 and S2.

**TABLE 2:** Multivariable logistic regression analysis of predictors for achieving SALT ≤ 20 at Week 36 among patients treated with baricitinib.

	Adjusted model	
	OR (95% CI)	*p* value
Sex (female)	0.41 (0.057 to 2.22)	0.309
Time from initial onset of AA to treatment initiation < 2 years	0.11 (0.006 to 0.997)	0.049
Baseline SALT score < 95	0.15 (0.014 to 1.062)	0.057
Complete eyebrow loss at baseline (ClinRO eyebrow score = 3)	0.05 (0.001 to 0.759)	0.029
Complete eyelash loss at baseline (ClinRO eyelash score = 3)	0.09 (0.002 to 1.117)	0.062
History of intravenous corticosteroid pulse therapy	0.61 (0.061 to 4.829)	0.641

*Note:* This table presents the results of a multivariable logistic regression model evaluating baseline predictors associated with achieving a SALT ≤ 20 response at Week 36. Variables were selected based on clinical relevance and evidence from phase III trials. Shorter disease duration (< 2 years) and complete eyebrow hair loss (ClinRO score 3) at baseline were identified as independent predictors of favorable response. These findings suggest that disease chronicity and eyebrow and eyelash hair involvement patterns may influence responsiveness to baricitinib in real‐world clinical settings. Odds ratios < 1 indicate a higher likelihood of achieving a SALT ≤ 20 response.

Abbreviations: AA, alopecia areata; CI, confidence interval; ClinRO, clinician‐reported outcome; OR, odds ratio; SALT, severity of alopecia tool.

### 3.7 Eyebrow and Eyelash Regrowth

**FIGURE 2:**
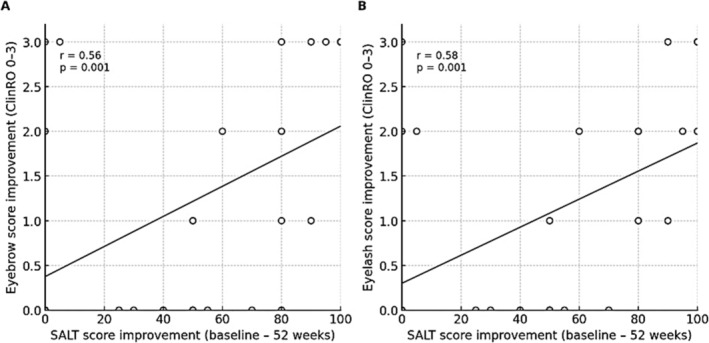
Association between scalp hair regrowth and eyebrow/eyelash recovery during baricitinib therapy. This figure demonstrates the relationship between scalp hair regrowth, measured by SALT score improvement, and recovery of eyebrow/eyelash hair based on clinician‐reported outcome (ClinRO) scores. Patients achieving substantial scalp recovery typically showed parallel improvement in eyebrow and eyelash involvement. In this cohort, complete eyebrow loss at baseline did not preclude scalp response, and several patients with ClinRO eyebrow score 3 achieved SALT ≤ 20. These findings support a clinically relevant association between scalp, eyebrow and eyelash hair regrowth, implying that early eyebrow/eyelash improvement may serve as a favorable response indicator. Correlations between changes in SALT and ClinRO scores were evaluated using Spearman's rank correlation coefficient. ClinRO, clinician‐reported outcome; SALT, severity of Alopecia tool.

We next examined the relationship between scalp hair regrowth and recovery of eyebrow and eyelash hair during baricitinib therapy (Figure 2). Overall, improvement in SALT scores tended to parallel improvements in eyebrow and eyelash ClinRO scores, indicating that hair recovery frequently occurred across multiple anatomical sites during treatment. Many patients who achieved substantial scalp regrowth also demonstrated partial or complete restoration of eyebrow and/or eyelash hair. Several patients with complete eyebrow loss at treatment initiation subsequently achieved clinically meaningful scalp regrowth (SALT ≤ 20).

### 3.8 Outcomes After Treatment Discontinuation or Reduction

An important practical question in managing AA with JAK inhibitors is what happens if treatment is stopped or the dose is reduced after a good response is achieved. In our baricitinib‐treated patients, 21 of the 50 patients had achieved a SALT ≤ 20 response by Week 36. We followed the post‐36‐week course of those who responded. Among these responders, four patients (median age, 44 years; range, 32–50 years), all of whom were female, elected to discontinue baricitinib completely after achieving marked hair regrowth, for various reasons including patient preference and external circumstances. Unfortunately, all four of these patients experienced a relapse of alopecia after stopping treatment. Notably, one patient remained in remission for about 60 weeks off therapy but then had a relapse that appeared to be triggered by an influenza infection. In the remaining three patients, in whom no clear external trigger for relapse was identified, regrown hair was lost substantially earlier, with a mean time to relapse of 14.7 weeks after treatment discontinuation (12, 13, and 19 weeks). This suggests that in cases where baricitinib must be stopped, there is a particularly high risk of hair loss returning within about 3 months, and careful observation is needed especially in that timeframe. For example, as shown in Figure S1, a representative patient who tapered baricitinib from 4 mg to 2 mg developed visible patchy recurrence within 3 months visually illustrating the relapse patterns observed in this cohort.

After achieving a SALT ≤ 20 response, 13 patients underwent dose reduction from 4 mg/day to 2 mg/day. Among the 12 evaluable cases, relapse of alopecia was confirmed in six patients, resulting in a relapse rate of 50% (Table 3). The mean time to relapse was 24.2 weeks after tapering (range, 12–52 weeks). One patient transferred to another institution shortly after dose reduction and was therefore not assessable for relapse evaluation (Case 12). These findings indicate that while dose tapering may allow sustained regrowth in selected patients, half of the evaluable cases experienced recurrence within approximately 6 months, underscoring the need for careful clinical monitoring and timely re‐escalation or alternative treatment strategies when signs of relapse emerge.

**TABLE 3:** Individual patient‐level outcomes after dose reduction of baricitinib to 2 mg/day in severe alopecia areata.

Case	Age (years)	Sex	Baseline SALT score	Duration from initiation to dose reduction (weeks)	Relapse	Time to relapse (weeks)	Re‐escalation to 4 mg/day	Outcome	Observation Period After Dose Reduction (weeks)
1	76	F	100	48	Yes	12	Yes	Responder	46
2	39	F	50	25	Yes	15	Yes	Non‐responder	59
3	17	M	50	45	Yes	16	Yes	Responder	69
4	49	M	100	71	Yes	22	Yes	Non‐responder	93
5	48	F	70	49	Yes	28	Yes	Responder	56
6	43	F	100	76	Yes	52	Yes	Maintenance of response	158
7	27	M	80	78	No	N/A	N/A	Maintenance of response	89
8	53	F	50	93	No	N/A	N/A	Maintenance of response	62
9	41	F	50	85, 133	No	N/A	N/A	Responder	65
10	29	M	50	124	No	N/A	N/A	Maintenance of response	8
11	51	M	100	83	No	N/A	N/A	Responder	21
12	50	F	50	26	N/A	N/A	N/A	Transferred to another institution	68
13	76	F	80	42	No	N/A	N/A	Maintenance of response	39

*Note:* This table details demographic characteristics, baseline disease severity, timing of tapering, relapse occurrence, time to relapse, management after relapse, and follow‐up duration in 13 patients who achieved SALT ≤ 20 on baricitinib 4 mg/day and subsequently reduced the dose to 2 mg/day. Among 12 evaluable cases, 6 patients (50%) relapsed within a mean of 24.2 weeks (range: 12–52). One patient (Case 12) transferred shortly after tapering and was not assessable. These real‐world data characterize relapse patterns and durability of response after dose reduction, underscoring the need for close surveillance and timely re‐escalation in patients showing early signs of recurrence. Case 9 underwent two separate dose‐reduction attempts; Case 12 was classified as N/A due to transfer and loss to follow‐up.

Abbreviations: N/A, not assessable; SALT, severity of Alopecia tool.

### 3.9 Ritlecitinib Efficacy and Prior JAK Inhibitor Exposure

**FIGURE 3:**
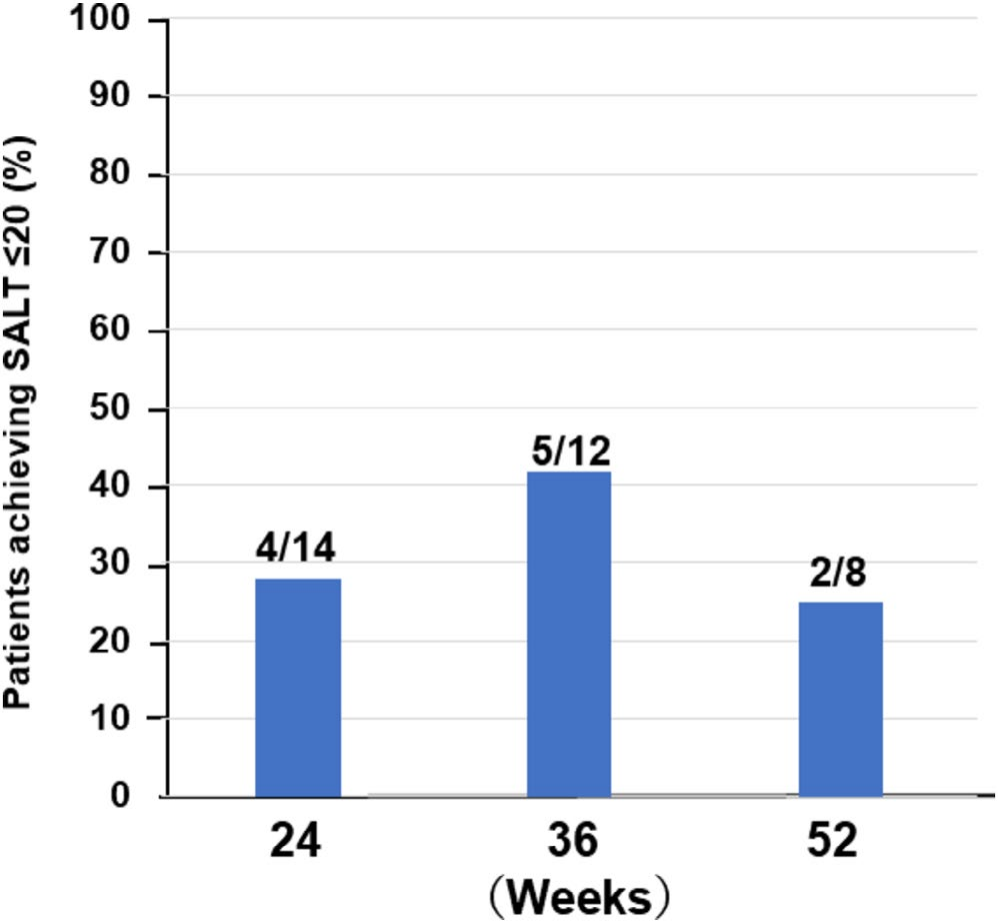
Proportion of patients achieving SALT ≤ 20 during ritlecitinib therapy. This figure shows the treatment response trajectory in patients with severe alopecia areata treated with ritlecitinib. The proportion achieving SALT ≤ 20 (≥ 80% scalp coverage) is presented at Weeks 24, 36, and 52. Among evaluable patients, response rates were 28.6% (4/14) at Week 24, 41.7% (5/12) at Week 36, and 25.0% (2/8) at Week 52. Although the sample size decreased over time due to more recent drug introduction, outcomes suggest that ritlecitinib induces substantial regrowth in approximately 30%–40% of patients in real‐world settings. Despite limited long‐term follow‐up, the efficacy trajectory appears numerically similar; however, cross‐treatment comparisons should be interpreted cautiously.

In the ritlecitinib group, fewer patients had reached the 36‐week time point at the time of analysis (due to later introduction of this therapy). Among those with follow‐up data, 28.6% (4/14 patients) achieved SALT ≤ 20 at Week 24, and 41.7% (5/12 patients) did so by Week 36. By Week 52, in the subset of patients who were still being followed, 25.0% (2/8) had achieved a SALT ≤ 20 response (Figure 3). Although the sample size is small, these figures suggest that ritlecitinib can induce significant hair regrowth in roughly 30%–40% of patients. Cross‐treatment comparisons should be interpreted cautiously given differences in baseline severity and prior JAK inhibitor exposure.

**FIGURE 4:**
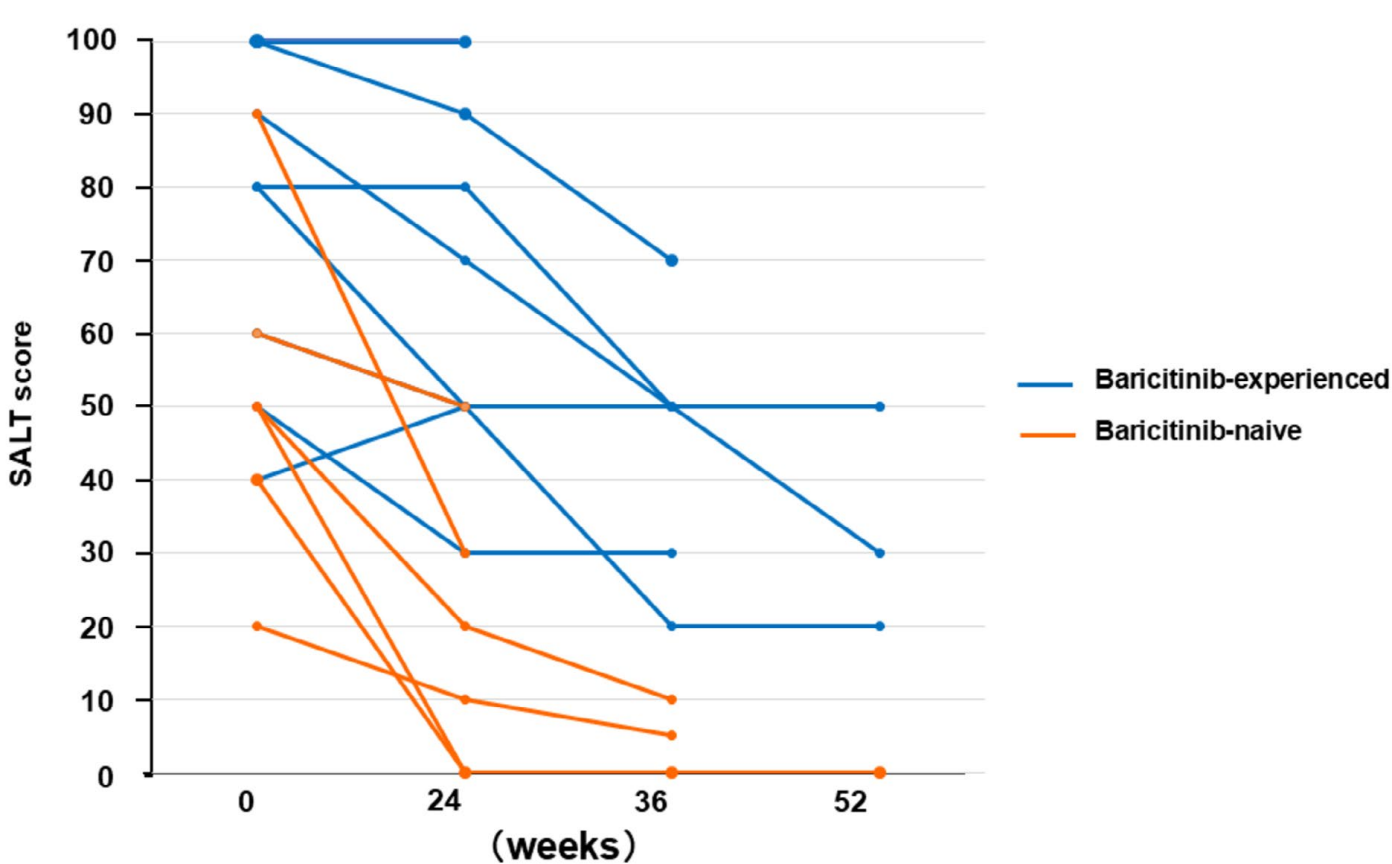
Comparison of ritlecitinib response according to prior baricitinib exposure. This figure compares treatment response patterns between baricitinib‐naïve and baricitinib‐experienced patients receiving ritlecitinib. Response is expressed as the proportion achieving SALT ≤ 20 (≥ 80% scalp regrowth) over time. Baricitinib‐naïve patients tended to show earlier and more robust regrowth, whereas baricitinib‐experienced patients demonstrated a slower and less consistent response. These findings suggest that prior JAK inhibitor exposure may influence the kinetics and magnitude of response to ritlecitinib. Although interpretation is limited by small sample size, results raise the possibility that ritlecitinib may be particularly beneficial as a first‐line JAK inhibitor, while still offering therapeutic value as a second‐line agent in selected cases.

We explored whether prior treatment with baricitinib influenced the response to ritlecitinib, given that 60% of the ritlecitinib group had a history of baricitinib use. Although our sample size was small, we observed a trend suggesting that patients without previous baricitinib treatment tended to respond to ritlecitinib more quickly and robustly. Those who were JAK‐inhibitor naïve sometimes showed hair regrowth as early as within 24 weeks of starting ritlecitinib (Figure 4). In contrast, patients who had prior exposure to baricitinib (and presumably had not fully responded to it) tended to require a longer duration on ritlecitinib to achieve regrowth, if they responded at all.

### 3.10 Adverse Events

Both baricitinib and ritlecitinib were generally well tolerated in this cohort, and most patients were able to continue therapy without interruption. In the baricitinib group (*n* = 50), the safety profile observed was consistent with known side effects of baricitinib as reported in other indications (e.g., rheumatoid arthritis and atopic dermatitis) (Table 4).

**TABLE 4:** Adverse events observed during treatment with baricitinib or ritlecitinib in severe alopecia areata.

Adverse events	Baricitinib (*N* = 50)	Ritlecitinib (*N* = 15)
Acne/Folliculitis	14.0% (7)	6.7% (1)
Elevated AST/ALT	8.0% (4)	6.7% (1)
Stomatitis/Oral inflammation	4.0% (2)	0% (0)
Gastrointestinal symptoms (nausea, constipation)	4.0% (2)	0% (0)
Elevated creatine kinase (CK)	4.0% (2)	0% (0)
Herpes simplex/Genital herpes	2.0% (1)	0% (0)
Pharyngitis/Sore throat	2.0% (1)	0% (0)
Ophthalmologic events (subconjunctival hemorrhage)	2.0% (1)	0% (0)
Dyslipidemia	2.0% (1)	0% (0)
Hyperkalemia	2.0% (1)	0% (0)
Serious adverse events requiring discontinuation	4.0% (2) (drug‐induced liver injury)	0% (0)
Total patients with ≥ 1 adverse event	16.0% (8)	13.3% (2)

*Note:* This table summarizes treatment‐emergent adverse events (AEs) in patients with severe alopecia areata treated with baricitinib or ritlecitinib. AEs are presented by system organ class and reported as number and percentage of affected patients in each group. In the baricitinib cohort, the most common AEs were acne/folliculitis and transient liver enzyme elevation; two patients discontinued therapy due to drug‐induced liver injury, both resolving after withdrawal. In the ritlecitinib group, AEs were infrequent and no discontinuations occurred. No thromboembolic events, serious infections, or unexpected safety signals were observed in either cohort during follow‐up, supporting the overall tolerability of both JAK inhibitors in real‐world management of severe alopecia areata. Data are presented as the number (percentage) of patients.

Abbreviations: AST, aspartate aminotransferase; ALT, alanine aminotransferase; CK, creatine kinase.

The most common adverse effects were acne or folliculitis, which occurred in 7 patients. Transient elevations in liver enzymes were seen in 4 patients. Other side effects included oral mucosal inflammation (stomatitis) in 2 patients, gastrointestinal discomfort or constipation in 2 patients, asymptomatic elevation of creatine kinase in 2 patients, genital herpes in 1 patient, sore throat (pharyngitis) in 1 patient, a case of subconjunctival hemorrhage (recorded as “corneal hemorrhage”) in 1 patient, a case of dyslipidemia in 1 patient, and one instance of hyperkalemia. Importantly, these adverse events were mostly mild. Out of the 4 patients who had liver function test elevations, only 2 required discontinuation of baricitinib; in those two cases, the liver enzyme abnormalities were classified as drug‐induced liver injury per Japanese hepatology criteria and resolved after switching to a different therapy. No thromboembolic events or other serious adverse events were observed in the baricitinib group, and the majority of patients were able to maintain therapy with proper monitoring.

In the ritlecitinib group (*n* = 15), adverse events were infrequent. We recorded only one patient with acne/folliculitis and one patient with abnormal liver enzymes during ritlecitinib treatment. No patient in the ritlecitinib group had to discontinue therapy due to side effects. Although our follow‐up for ritlecitinib was shorter, no serious adverse events (such as severe infections, thrombosis, or other major complications) were noted in the observed period.

## 4 Discussion

In this real‐world study of refractory AA, both baricitinib and ritlecitinib proved to be effective treatment options, inducing substantial hair regrowth in a significant proportion of patients while demonstrating a generally favorable safety profile. At 36 weeks, 42% of patients on baricitinib 4 mg daily achieved a SALT ≤ 20 response (≥ 80% scalp hair regrowth), which is in line with the response rates reported in phase 3 trials [6]. This concordance with controlled trial efficacy reinforces the robustness of baricitinib's effect in a real‐world setting. Moreover, many responders in our cohort were able to maintain or even improve their hair regrowth with continued therapy through 52 weeks, suggesting that longer treatment can further increase the proportion of patients achieving substantial regrowth. This observation is consistent with extended trial data indicating that the probability of response rises with ongoing therapy (e.g., ~40%–64% of patients on baricitinib 4 mg had SALT 20 or less between Week 36 and Week 52 in 3‐year analyses) [10]. Notably, our real‐world baricitinib outcomes are also comparable to other recent cohorts: a Korean multicenter study (117 patients) reported ~55% achieving SALT ≤ 20 by Week 36, [11] and a Japanese single‐center series (70 patients) similarly demonstrated a 41% SALT ≤ 20 response at Week 36 [12]. In a separate long‐term follow‐up from the same institution, 36 patients continued baricitinib therapy for 104 weeks, of whom 55.6% maintained a SALT ≤ 20 response at two years [13]. Together, these data affirm that baricitinib can induce clinically meaningful regrowth in approximately half of adults with severe AA within 6–12 months in real‐world practice, with a subset achieving durable long‐term control when therapy is maintained.

Ritlecitinib, a selective JAK3/TEC inhibitor, also demonstrated encouraging real‐world effectiveness in our study. By 36 weeks, 42.0% of ritlecitinib‐treated patients achieved ≥ 80% scalp hair regrowth, a response rate numerically higher than that reported at 24 weeks in the drug's pivotal trial (23.0% on 50 mg) [7]. This difference likely reflects the longer observation period in our cohort, as some patients who were non‐responders at earlier time points went on to show significant regrowth by 36 weeks. Indeed, the need for extended treatment is supported by the integrated analysis of the ALLEGRO phase IIb/III and long‐term phase III trials, which demonstrated that continued ritlecitinib therapy beyond Week 24 resulted in progressive improvement in hair density not only on the scalp but also in the eyebrows and eyelashes [14]. Our findings also suggest that ritlecitinib's efficacy is at least comparable to baricitinib's in a real‐world setting, with overlapping 36‐week response rates. However, it should be emphasized that the two treatment cohorts were not directly comparable at baseline, as the ritlecitinib group included a higher proportion of baricitinib‐refractory patients and exhibited greater clinical heterogeneity, which may have influenced observed response rates and precludes a strict head‐to‐head efficacy interpretation. However, patient heterogeneity and prior treatment history must be considered. In our cohort, 60% of ritlecitinib patients had previously used baricitinib (with inadequate results), raising the question of whether prior JAK inhibitor exposure affects subsequent response. We observed a trend whereby JAK‐inhibitor–naïve patients tended to respond faster and more robustly to ritlecitinib–sometimes achieving visible regrowth by 3–6 months‐whereas those who had failed baricitinib often required a longer ritlecitinib treatment duration to attain regrowth (if at all). This aligns with a recent report by Huang et al., who found that prior JAK inhibitor exposure was an independent predictor of reduced early response to ritlecitinib [15]. These findings are further supported by other recent real‐world studies. Okazaki et al. reported similar response patterns in a Japanese cohort, demonstrating markedly higher SALT50 and SALT75 achievement among JAK inhibitor–naïve patients compared with those previously treated with baricitinib, reinforcing the impact of prior JAK exposure on treatment responsiveness [16]. In addition, Chen et al. documented consistent short‐term efficacy and favorable safety of ritlecitinib across a broader clinical population, providing complementary evidence that the drug performs reliably outside of controlled trials [17]. It appears that while switching from one JAK inhibitor to another can yield benefits for some non‐responders, these patients might experience a delayed or attenuated response. Our real‐world cases included several individuals who, despite not responding to baricitinib, eventually achieved clinically meaningful regrowth on ritlecitinib after prolonged therapy (beyond 6 months). This outcome is encouraging, as it indicates that lack of response to one JAK inhibitor does not preclude responsiveness to another–potentially due to differences in kinase selectivity or patient‐specific immunologic pathways. Nevertheless, given our small sample size, we interpret these switching outcomes with caution. Prospective studies or larger registries are needed to determine the efficacy of sequential JAK inhibitor therapy and to identify which patients are most likely to benefit from switching (or combination approaches) when an initial JAK inhibitor fails.

An important aim of this study was to identify clinical factors predictive of treatment success. In the baricitinib cohort, we found two independent predictors of favorable response at 36 weeks: shorter disease duration (< 2 years from AA onset) and complete eyebrow hair loss at baseline. Interestingly, complete eyebrow loss at baseline was independently associated with a higher likelihood of achieving SALT ≤ 20 in our multivariable analysis. Although this finding may appear counterintuitive, as complete eyebrow loss is generally considered a marker of extensive disease, it suggests that even patients with severe clinical presentation may retain substantial therapeutic responsiveness to JAK inhibition. One possible explanation is that complete eyebrow loss in our cohort frequently coincided with relatively shorter disease duration, reflecting an active inflammatory state rather than long‐standing, treatment‐refractory alopecia. Thus, eyebrow involvement should not be interpreted solely as a negative prognostic indicator. Nevertheless, this observation requires cautious interpretation and validation in larger prospective cohorts. We selected the < 2 year threshold based on both clinical rationale and exploratory analyses within our cohort, as it captured very recent‐onset disease and demonstrated greater discriminatory performance than longer cut‐offs reported previously. The association between shorter disease duration and better outcomes is consistent with prior observations–patients with more recent onset of AA may have a higher proportion of reversible, active hair follicles that can be rescued by immunomodulation, whereas long‐standing disease might involve more irreversible follicular damage or stem cell depletion. A recent Japanese multivariate analysis of 70 baricitinib‐treated patients similarly noted that disease duration ≤ 4 years was a strong predictor of achieving SALT 20 regrowth [12]. Likewise, a systematic review highlighted that patients with longer current episodes and higher baseline severity are at increased risk of inadequate response to JAK inhibitors [18]. Our findings reinforce the notion that earlier intervention in the course of AA could yield better regrowth outcomes, and that prolonged untreated disease may be a negative prognostic factor. Importantly, exploratory analyses using alternative clinical endpoints (SALT50/SALT75 relative improvement) showed broadly consistent predictor patterns, supporting the robustness of our findings across different definitions of treatment response.

Eyebrow involvement is usually regarded as a marker of severe, extensive AA and has often been associated with poorer prognosis in natural history studies [19]. Intuitively, one might assume that patients who have lost all eyebrow hair (indicative of a more aggressive autoimmune attack) would respond less favorably. However, in our cohort, those with ClinRO eyebrow score 3 (no visible eyebrows) were actually more likely to achieve significant scalp regrowth on baricitinib by Week 36. One possible explanation is that complete eyebrow loss in these cases signified highly active immune inflammation that was nonetheless acutely responsive to JAK inhibition, as opposed to a smoldering chronic process. In other words, these patients may represent a phenotype of AA with robust inflammatory involvement (capable of affecting eyebrows) but without irreversible destruction of hair follicles. With potent systemic immunosuppression via JAK1/2 blockade, such patients exhibited a “high reversibility” profile–rapidly regrowing hair once the immune attack was quelled. This interpretation is indirectly supported by prior research showing that markers of active inflammation predict treatment responsiveness. For example, a trial of tofacitinib in severe AA found that the presence of peri‐bulbar inflammatory infiltrates on baseline scalp biopsy correlated with positive hair regrowth outcomes [20], whereas patients lacking signs of active inflammation (perhaps reflecting a burned‐out disease state) were less likely to respond. It is conceivable that complete eyebrow loss in some patients is a transient manifestation of intense inflammation (which can be reversed), whereas in others it may reflect a chronic refractory state. We did observe that eyebrow and eyelash regrowth tended to parallel scalp regrowth in responders: many patients who regrew scalp hair also had improvement in their eyebrows and eyelashes over time, indicating a synchronized recovery across hair‐bearing sites. On the other hand, some of the poorest scalp responders in our series showed no regrowth of brows or lashes. These patterns suggest that “eyebrow involvement” actually captures heterogeneous scenarios–either an aggressive yet JAK‐responsive disease or a very severe, resistant disease. From a practical standpoint, early improvement in eyebrow or eyelash hair during therapy could serve as a harbinger of a favorable overall response, as has been proposed in the literature [21]. Further investigation in larger cohorts is warranted to clarify the prognostic implications of specific body hair involvement and to determine whether features like eyebrow loss should influence treatment decisions (e.g., starting JAK inhibitors sooner in those patients).

Notably, other factors that have been proposed as response predictors were not significant in our multivariable analysis. For instance, prior studies suggested that female sex, lower baseline SALT score (less extensive hair loss), and history of systemic corticosteroid therapy (e.g., intravenous methylprednisolone pulse treatments) might predict better responses to JAK inhibitors [12]. In our dataset, trends in these factors were observed (e.g., numerically higher response rates in females and in those with baseline SALT < 95), but they did not reach statistical significance, potentially due to limited sample size or confounding. Alopecia totalis/universalis was also not independently predictive in our study after controlling for duration and other variables. Nevertheless, it is intuitive that patients with milder baseline scalp involvement (patchy AA) might have an easier time regrowing hair than those with complete scalp loss; indeed, the Korean real‐world study noted that patients with baseline SALT ≤ 50 had higher rates of achieving SALT 75/90/100 than those who started with SALT > 50 [11]. Similarly, an Italian 48‐week study identified several predictors of treatment response to baricitinib, reporting that responders were more often female, younger, and had shorter disease duration, and that patients with severe (but not very severe) alopecia areata achieved SALT ≤ 20 more rapidly–showing a higher proportion of early responders compared with the most severe group (*p* = 0.035), whereas duration of the current episode did not significantly influence time to response [22]. These discrepancies highlight the variability in patient populations and the multifactorial nature of treatment response in AA. It is likely that a combination of factors–disease duration, extent, pattern, patient sex/age, immune profile, etc.–collectively influences outcomes, and no single predictor is absolute. Developing a predictive model or scoring system (potentially incorporating biomarkers) remains an important goal for future research, as it could help personalize JAK inhibitor therapy by identifying those most likely to benefit.

Our extended follow‐up allowed us to examine the course of AA after treatment discontinuation or dose tapering–a scenario highly relevant to real‐world management. The data strongly illustrate the chronic, relapsing nature of AA and the challenge of maintaining remission off therapy. In our cohort, all four responders who stopped baricitinib eventually relapsed, with substantial scalp hair loss typically recurring within about 3–4 months after drug cessation (mean time to relapse ~14.7 weeks). These observations are consistent with the BRAVE‐AA1 randomized withdrawal trial, in which discontinuation of baricitinib led to rapid loss of treatment benefit, with 0% of patients relapsing at Week 4, 10%–11% by Week 8, and approximately 80% losing clinical benefit by long‐term follow‐up (Week 152), whereas only 7% of those who continued baricitinib maintained relapse‐free status at the same timepoint [23]. Our real‐world relapse timing is likewise consistent with earlier observations from tofacitinib: many patients begin shedding hair again within ~2–3 months of stopping JAK inhibitor therapy. A meta‐analysis of observational studies reported that approximately one‐quarter of patients experienced relapse, most of which occurred following discontinuation of tofacitinib, [24] highlighting that treatment withdrawal is a major driver of disease recurrence; although pooled relapse risk has been estimated around 54% in real‐world settings, the likelihood of recurrence may approach universal levels over prolonged timelines, particularly in severe or chronic AA. Together, these data make it clear that continuous therapy is usually required to sustain hair regrowth in severe AA. In other words, AA behaves as a relapsing–remitting disease, and JAK inhibitors–much like other treatments for chronic autoimmune conditions–appear to control the disease only while actively administered, without inducing a permanent cure in most cases.

Interestingly, we observed that reducing the baricitinib dose to 2 mg/day (half the standard dose) delayed the return of alopecia compared to complete cessation, but relapse still occurred in roughly half of those tapered. Among patients who stepped down to 2 mg after achieving good regrowth on 4 mg, 50% relapsed, with a longer average time to relapse (~24 weeks, or about 6 months). The remainder maintained their hair at the lower dose during the observed period. This suggests that a subset of patients can be “dose dialed” to a lower maintenance dose (potentially mitigating long‐term risks), but even with dose tapering, vigilance is needed. While controlled data on dose reduction in AA are limited, our findings parallel a concept seen in other immune‐mediated diseases: some patients in remission can be kept on a reduced dose and do well for some time, but many will eventually flare if the dose is insufficient. Although controlled data on dose reduction for AA remain limited, our observations parallel concepts well recognized in other immune‐mediated inflammatory diseases: a subset of patients in remission can maintain disease control on a lower maintenance dose, whereas many ultimately relapse if suppression becomes subtherapeutic. Importantly, recently published long‐term extension data addressed this issue more explicitly: at Week 52, 86 of 234 patients (36.8%) receiving baricitinib 4 mg were eligible for dose reduction, of whom 44 continued 4 mg and 42 tapered to 2 mg [25]. By Week 152, clinical response was maintained in 39/44 (88.6%) continuing 4 mg and 24/41 (58.5%) who tapered, and notably, loss of efficacy was uncommon in dose‐reduced patients who had sustained deep response (SALT ≤ 5) at Week 52. These findings–together with our real‐world outcome where half of dose‐reduced patients relapsed within ~6 months–suggest that > 50% of patients can maintain response after de‐escalation, but the risk of relapse remains meaningful. Durable treatment response and/or near‐complete regrowth at the time of tapering may therefore increase the likelihood of remission maintenance following dose reduction.

From a management perspective, these results highlight that discontinuation of JAK inhibitor therapy in severe AA should be approached with caution. The decision to stop or taper should take into account the patient's disease history (e.g., if AA flares have been frequent and severe in the past, one might anticipate a rapid relapse). When therapy must be paused–for example, due to pregnancy, surgery, intercurrent infection, or insurance issues–patients should be made aware of the high likelihood of relapse and the need to resume treatment quickly if hair loss recurs. Likewise, if tapering is attempted, a structured follow‐up schedule (e.g., monthly examinations for 6 months) is prudent. Encouragingly, most patients who relapse are able to regain their regrowth upon restarting the medication. In the BRAVE withdrawal study, over 80% of relapsing patients who resumed baricitinib were able to recapture their SALT 20 response [23]. We also observed this pattern anecdotally in our clinic: patients who lost hair after stopping baricitinib often responded again when the drug was reintroduced (data not shown). This indicates that relapse after stopping is not due to tachyphylaxis or permanent drug failure, but rather the disease reasserting itself when pharmacologic control is removed. Altogether, our findings and the literature suggest that for patients with refractory, long‐standing AA, JAK inhibitors function as maintenance therapy–much like treatments for other chronic autoimmune diseases–and continuous or long‐term use is necessary to maintain remission.

Safety is a key consideration for any long‐term therapy. In our study, baricitinib and ritlecitinib were generally well tolerated, and no new or unexpected safety signals emerged over a combined patient‐years of observation. The adverse events (AEs) we observed were mostly mild to moderate in severity and could be managed with standard interventions or brief treatment interruptions. The most common AEs with baricitinib were acneiform eruptions and folliculitis, transient elevations in liver enzymes, aphthous ulcers, and mild infections (e.g., pharyngitis, herpes labialis) – all of which have been reported in prior baricitinib trials in AA and in its approved indications (rheumatoid arthritis and atopic dermatitis) [26, 27]. The incidence of these AEs in our cohort was comparable to that in clinical trials and other real‐world studies. Importantly, we did not encounter any thromboembolic events, serious infections, malignancies, or other grade 3/4 toxicities. Our findings provide reassurance, consistent with the growing body of evidence that JAK inhibitors can be used safely in AA with proper patient selection and monitoring. Ritlecitinib, being a newer agent, had a smaller exposure in our study (15 patients with shorter follow‐up) but showed a benign short‐term safety profile. Only two mild AEs (folliculitis and transient hepatic enzyme elevation) were recorded in the ritlecitinib group, and no patient discontinued ritlecitinib due to side effects. This aligns with ritlecitinib's trial data, where no significant safety differences from placebo were noted apart from mild acne and headache. Long‐term data up to 2–3 years have likewise indicated that ritlecitinib remains well tolerated with no evidence of cumulative toxicity.

In conclusion, this real‐world study demonstrates that nearly half of adults with severe, treatment‐refractory alopecia areata achieved clinically meaningful scalp hair regrowth with either baricitinib or ritlecitinib. We identified shorter disease duration and specific hair‐loss patterns–particularly complete eyebrow involvement at baseline–as factors associated with a more favorable response to baricitinib, underscoring the importance of patient selection and timely initiation of therapy. Relapse was frequent following treatment discontinuation or dose reduction, typically occurring within approximately 3 months after cessation and within 6 months after tapering, indicating that continuous therapy or vigilant post‐deescalation monitoring is required to maintain regrowth. These findings provide practical guidance for long‐term management strategies and support the integration of JAK inhibitors as central therapeutic options in routine AA care.

## Funding

The authors have nothing to report.

## Conflicts of Interest

Saori Takamura has received lecture fees from Maruho, Sun Pharma, AbbVie, Kaken Pharmaceutical, UCB Japan, Torii Pharmaceutical, BioCryst, Kyowa Kirin, Bristol Myers Squibb, Eli Lilly Japan, CSL Behring, LEO Pharma, Sanofi, Pfizer, Janssen Pharmaceutical, Takeda Pharmaceutical, and Amgen. Tomoo Fukuda has received lecture fees from AbbVie, Sato Pharmaceutical, Eli Lilly Japan, CSL Behring, Takeda Pharmaceutical, Maruho, and Kaken Pharmaceutical, and scholarship donations from the Maruho‐Takagi Foundation for Dermatology Research. Rina Hayashi declares no conflicts of interest.

## Supporting information


**Figure S1:** jde70241‐sup‐0001‐FigureS1.tif.
The figure includes the following panels:
(A) Baseline prior to initiation of baricitinib 4 mg/day, demonstrating extensive scalp hair loss.
(B) Week 36 during continuous treatment with baricitinib 4 mg/day, showing near‐complete scalp hair regrowth.
(C) Three months after dose reduction to 2 mg/day, demonstrating recurrence of patchy scalp hair loss.
These images illustrate the risk and clinical extent of relapse following dose tapering in responders and highlight the importance of appropriate maintenance therapy in severe alopecia areata.
Written informed consent for publication of these clinical photographs was obtained from the patient.

**Table S1:** Multivariable Logistic Regression Analysis of Predictors for Achieving SALT50 at Week 36 in Baricitinib‐Treated Patients.
This supplementary table presents the results of a multivariable logistic regression analysis evaluating baseline clinical predictors associated with achieving a ≥ 50% relative improvement in scalp hair regrowth (SALT50) at Week 36 among patients treated with baricitinib. The dependent variable was achievement of SALT50, and independent variables included sex, disease duration from initial AA onset (< 2 years vs. ≥ 2 years), baseline SALT score (< 95 vs. ≥ 95), complete eyebrow loss (ClinRO score = 3), complete eyelash loss (ClinRO score = 3), and history of intravenous corticosteroid pulse therapy.
Adjusted odds ratios (ORs) with 95% confidence intervals (CIs) are shown. In this model, a baseline SALT score < 95 was significantly associated with achievement of SALT50 at Week 36 (*p* = 0.033). Odds ratios < 1 indicate a higher likelihood of achieving the SALT50 endpoint. AA, Alopecia areata; CI, Confidence Interval; ClinRO, Clinician‐Reported Outcome; OR, Odds Ratio; SALT, Severity of Alopecia Tool.

**Table S2:** Multivariable Logistic Regression Analysis of Predictors for Achieving SALT75 (≥ 75% Relative Improvement from Baseline) at Week 36 in Baricitinib‐Treated Patients.
This supplementary table presents a multivariable logistic regression analysis identifying baseline predictors associated with achieving a ≥ 75% relative improvement in scalp hair regrowth (SALT75) at Week 36 in patients receiving baricitinib. The model incorporated clinically relevant baseline variables, including sex, disease duration from initial AA onset (< 2 years vs. ≥ 2 years), baseline SALT score (< 95 vs. ≥ 95), complete eyebrow loss (ClinRO score = 3), complete eyelash loss (ClinRO score = 3), and prior intravenous corticosteroid pulse therapy. Adjusted odds ratios (ORs) with 95% confidence intervals (CIs) are provided. In this model, female sex, baseline SALT score < 95, and complete eyebrow loss at baseline were significantly associated with achieving SALT75 at Week 36. Odds ratios < 1 indicate a higher likelihood of achieving the SALT75 endpoint. AA, alopecia areata; CI, confidence interval; ClinRO, clinician‐reported outcome; OR, odds ratio; SALT, severity of alopecia tool.

## Data Availability

The data that support the findings of this study are available on request from the corresponding author. The data are not publicly available due to privacy or ethical restrictions.
