# Supplementary figures and images for: Real‐World Outcomes of Baricitinib and Ritlecitinib in Refractory Alopecia Areata: Response Predictors and Relapse After Discontinuation or Dose Reduction
Source: J Dermatol. 2026 Mar 31;53(5):746–57. doi: 10.1111/1346-8138.70241 (PMC13150673; doi:10.1111/1346-8138.70241)

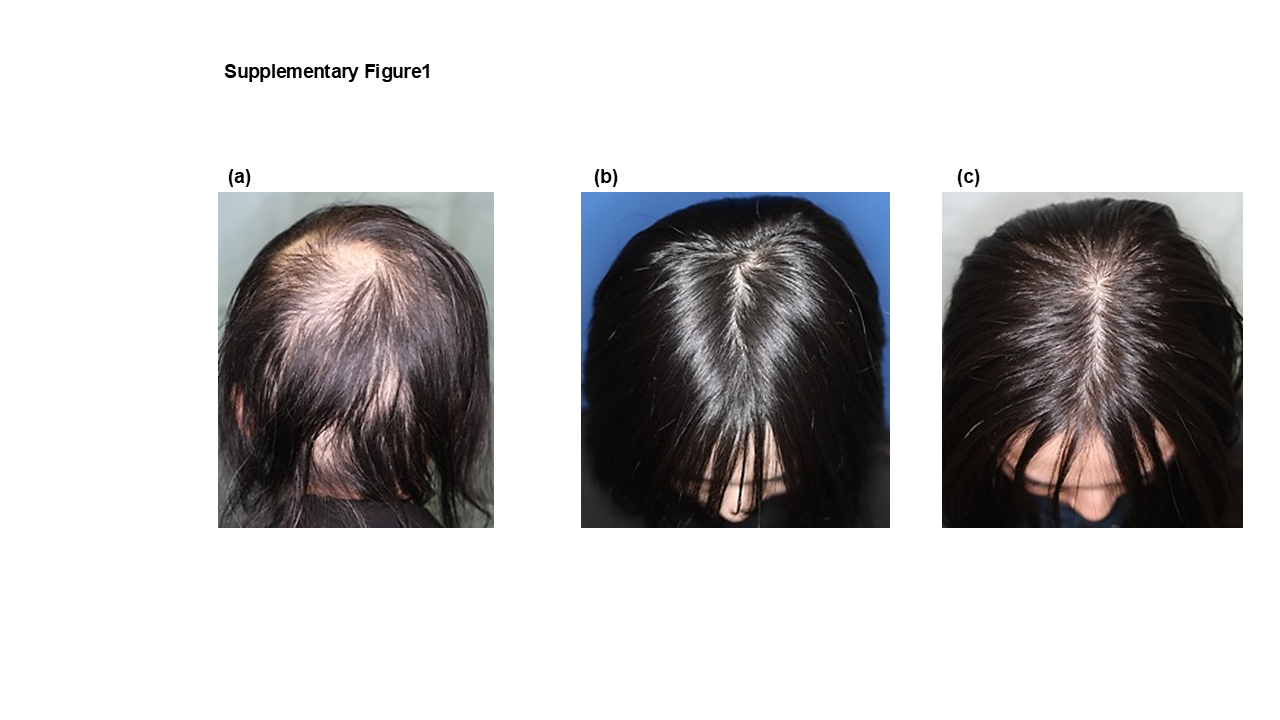

Supplement: Supplementary file 1 — Figure S1: jde70241‐sup‐0001‐FigureS1.tif. The figure includes the following panels: (A) Baseline prior to initiation of baricitinib 4 mg/day, demonstrating extensive scalp hair loss. (B) Week 36 during continuous treatment with baricitinib 4 mg/day, showing near‐complete scalp hair regrowth. (C) Three months after dose reduction to 2 mg/day, demonstrating recurrence of patchy scalp hair loss. These images illustrate the risk and clinical extent of relapse following dose tapering in responders and highlight the importance of appropriate maintenance therapy in severe alopecia areata. Written informed consent for publication of these clinical photographs was obtained from the patient. [file JDE-53-746-s002.tif]
