# Supplementary material for: Real‐World Outcomes of Baricitinib and Ritlecitinib in Refractory Alopecia Areata: Response Predictors and Relapse After Discontinuation or Dose Reduction
Source: J Dermatol. 2026 Mar 31;53(5):746–57. doi: 10.1111/1346-8138.70241 (PMC13150673; doi:10.1111/1346-8138.70241)
Supplement: Supplementary file 2 — Table S1: Multivariable Logistic Regression Analysis of Predictors for Achieving SALT50 at Week 36 in Baricitinib‐Treated Patients. This supplementary table presents the results of a multivariable logistic regression analysis evaluating baseline clinical predictors associated with achieving a ≥ 50% relative improvement in scalp hair regrowth (SALT50) at Week 36 among patients treated with baricitinib. The dependent variable was achievement of SALT50, and independent variables included sex, disease duration from initial AA onset (< 2 years vs. ≥ 2 years), baseline SALT score (< 95 vs. ≥ 95), complete eyebrow loss (ClinRO score = 3), complete eyelash loss (ClinRO score = 3), and history of intravenous corticosteroid pulse therapy. Adjusted odds ratios (ORs) with 95% confidence intervals (CIs) are shown. In this model, a baseline SALT score < 95 was significantly associated with achievement of SALT50 at Week 36 (p = 0.033). Odds ratios < 1 indicate a higher likelihood of achieving the SALT50 endpoint. AA, Alopecia areata; CI, Confidence Interval; ClinRO, Clinician‐Reported Outcome; OR, Odds Ratio; SALT, Severity of Alopecia Tool. [file JDE-53-746-s001.docx]

**Supplementary Table 1.** **Multivariable logistic regression analysis of predictors for achieving SALT 50 relative at Week 36 among patients treated with baricitinib.**

|  | Adjusted model | |
| --- | --- | --- |
|  | OR (95%CI) | *P* value |
| Sex **(female)** | 0.64 (0.164 to 2.47) | 0.518 |
| Time from initial onset of AA to treatment initiation **< 2 years** | 0.26 (0.035 to 1.256) | 0.097 |
| Baseline SALT score **<**95 | 0.21 (0.039 to 1.882) | 0.033 |
| Complete eyebrow loss at baseline  (ClinRO eyebrow score = 3) | 0.26 (0.061 to 1.053) | 0.059 |
| Complete eyelash loss at baseline  (ClinRO eyelash score = 3) | 0.47 (0.111 to 1.936) | 0.294 |
| History of intravenous corticosteroid pulse therapy | 0.62 (0.115 to 2.803) | 0.545 |

Abbreviations: OR, odds ratio; CI, confidence interval; AA, alopecia areata; SALT, severity of alopecia tool; ClinRO, clinician-reported outcome.

Odds ratios <1 indicate a higher likelihood of achieving a SALT 50 endpoint (relative improvement).
