# Supplementary material for: Real‐World Outcomes of Baricitinib and Ritlecitinib in Refractory Alopecia Areata: Response Predictors and Relapse After Discontinuation or Dose Reduction
Source: J Dermatol. 2026 Mar 31;53(5):746–57. doi: 10.1111/1346-8138.70241 (PMC13150673; doi:10.1111/1346-8138.70241)
Supplement: Supplementary file 3 — Table S2: Multivariable Logistic Regression Analysis of Predictors for Achieving SALT75 (≥ 75% Relative Improvement from Baseline) at Week 36 in Baricitinib‐Treated Patients. This supplementary table presents a multivariable logistic regression analysis identifying baseline predictors associated with achieving a ≥ 75% relative improvement in scalp hair regrowth (SALT75) at Week 36 in patients receiving baricitinib. The model incorporated clinically relevant baseline variables, including sex, disease duration from initial AA onset (< 2 years vs. ≥ 2 years), baseline SALT score (< 95 vs. ≥ 95), complete eyebrow loss (ClinRO score = 3), complete eyelash loss (ClinRO score = 3), and prior intravenous corticosteroid pulse therapy. Adjusted odds ratios (ORs) with 95% confidence intervals (CIs) are provided. In this model, female sex, baseline SALT score < 95, and complete eyebrow loss at baseline were significantly associated with achieving SALT75 at Week 36. Odds ratios < 1 indicate a higher likelihood of achieving the SALT75 endpoint. AA, alopecia areata; CI, confidence interval; ClinRO, clinician‐reported outcome; OR, odds ratio; SALT, severity of alopecia tool. [file JDE-53-746-s003.docx]

**Supplementary Table 2. Multivariable Logistic Regression Analysis of Predictors for Achieving SALT75 (≥75% Relative Improvement from Baseline) at Week 36 in Baricitinib-Treated Patients**

|  | Adjusted model | |
| --- | --- | --- |
|  | OR (95%CI) | *P* value |
| Sex **(female)** | 0.14 (0.018 to 0.690) | 0.015 |
| Time from initial onset of AA to treatment initiation **< 2 years** | 0.23 (0.011 to 1.496) | 0.133 |
| Baseline SALT score **<**95 | 0.21 (0.049 to 1.390) | 0.004 |
| Complete eyebrow loss at baseline  (ClinRO eyebrow score = 3) | 0.10 (0.012 to 0.498) | 0.005 |
| Complete eyelash loss at baseline  (ClinRO eyelash score = 3) | 0.27 (0.052 to 1.268) | 0.267 |
| History of intravenous corticosteroid pulse therapy | 0.26 (0.013 to 1.775) | 0.188 |

Abbreviations: OR, odds ratio; CI, confidence interval; AA, alopecia areata; SALT, severity of alopecia tool; ClinRO, clinician-reported outcome.

Odds ratios <1 indicate a higher likelihood of achieving a SALT 75 endpoint (relative improvement).
